# Efficacy of a New Non-drug Acne Therapy: Aloe Vera Gel Combined With Ultrasound and Soft Mask for the Treatment of Mild to Severe Facial Acne

**DOI:** 10.3389/fmed.2021.662640

**Published:** 2021-05-21

**Authors:** Hongyu Zhong, Xiang Li, Wanqi Zhang, Xiaoxiao Shen, Yuangang Lu, Hongli Li

**Affiliations:** ^1^Experimental Center of Basic Medicine, College of Basic Medical Science, Third Military Medical University, Chongqing, China; ^2^Department of Plastic Surgery and Cosmetic Surgery, Daping Hospital, Third Military Medical University, Chongqing, China

**Keywords:** acne (acne vulgaris), non-drug therapy, aloe vera gel, ultrasound, soft mask, hyperpigmentation

## Abstract

**Background:** Acne is a chronic disorder that affects almost 80% of adolescents and young adults, causing psychological and emotional distress. However, the current treatments for acne are either ineffective or have many side effects. This study was designed to confirm and objectively quantify the effect of a new non-drug combined therapy on acne.

**Methods:** This study innovatively utilized ultrasound, which enhanced the absorption of aloe vera gel, and soft mask to make a purely physical method without any drugs. In both the treatment group and control group, the number of papules/pustules and the area of hyperpigmented lesions were counted, and a smart mirror intelligent face system was used before and after the combined therapy. Alterations in the skin functional index were recorded and analyzed statistically.

**Results:** In the treatment group, the combined therapy significantly reduced the number of papules and the area of hyperpigmented lesions and improved skin roughness and local blood circulation. In the control group, there was no obvious improvement over 2 months.

**Conclusion:** This study suggests that the new non-drug combined therapy significantly improved acne, which provided experimental evidence and treatment guidance for patients with mild to severe acne, especially patients with moderate acne. This new therapy may possibly be an appropriate method for patients who seek topical treatments with mild side effects and low antibiotic resistance rates.

## Introduction

Acne is a chronic disorder of pilosebaceous units, which is characterized by comedones, inflammatory papules, pustules, cysts, and nodules, affecting almost 80% of adolescents and young adults (1, 2). Acne lesions can result in post-inflammatory hyperpigmentation (PIH) and scarring (3–5). Inflammation exists throughout the whole cycle of acne lesions, and PIH can cause psychological and emotional distress, leading to a great influence on healthy quality of life in affected individuals (6, 7). The current treatments include topical and systemic medications for moderate to severe acne (8–10). However, their curative effects are limited due to adverse effects and unsatisfactory efficacy. In addition, some medications remain controversial. For example, oral isotretinoin, the current mainstream systemic therapy, has acknowledged adverse reactions, including dizziness, nausea and gastrointestinal reaction. Additionally, its psychological effects and teratogenicity also upset the patients who received the treatment (11, 12). Moreover, the efficacy and evaluation of the combined therapy of photodynamic therapy and minocycline are limited because this treatment was only effective in reducing inflammatory and non-inflammatory lesions without therapeutic effects on PIH and overall skin conditions (13).

In recent years, there have been an increasing number of medicines and cosmetics made from aloe vera gel (AVG), the mucilaginous tissue in the center of the aloe vera leaf (14, 15). Its pharmacological functions include antibacterial, anti-inflammatory effects and wound healing promotion (16–18). A previous study suggested that AVG treatment in combination with tretinoin was effective in reducing non-inflammatory and inflammatory acne lesions. However, its adverse effects were also observed in more than 70% of treated patients, such as scaling, burning, and erythema (19). In addition, it has been reported that the high and low frequencies of ultrasound (US) waves have a role in disaggregating the horny layer and stimulating the absorption of the active compounds (20). This study was designed to evaluate the efficacy of AVG combined with US and external application of soft mask to offer an alternative to patients with mild to severe acne who seek topical treatments with mild side effects and low antibiotic resistance rates.

## Materials and Methods

### Study Subjects

Sixty-four patients (32 males, 32 females) aged 20–35 years with mild to severe facial acne and PIH were enrolled. All patients were randomly divided into two groups, the control group and treatment group, in this double-blind study. The treatment group contained 40 patients (24 males, 16 females), and the control group contained 24 patients (8 males, 16 females). There was no statistically significant difference in age between the two groups (*P* > 0.05). In the first week, skin care was carried out once a day. Then, it was changed three times a week for seven weeks. After treatment, the therapeutic efficacy was evaluated.

The inclusion criteria were as follows: (1) Patients who had not received hydroquinone, other depigmenting medication or light treatment at least 1 month before inclusion. (2) Patients with informed consent and active cooperation. (3) Patients without blood system diseases, such as blood clotting disorders. (4) Patients without serious underlying diseases, such as diabetes, hypertension, liver and kidney dysfunction. (5) Patients with normal immune function.

The exclusion criteria were as follows: (1) Patients who were allergic to any tool or drug used in the course of treatment. (2) Patients with incomplete treatment. (3) Patients with serious inflammation. (4) Pregnant women. (5) Patients with mental illness.

Our study was approved by the Ethics Committee of Third Military Medical University and was conducted in conformity with the Declaration of Helsinki. All patients provided written informed consent before enrollment in the study.

### Study Contents

The AVG (Perfect, China). The ultrasonic therapeutic apparatus (K-SKIN, 830, China). Powder of soft mask (MedSPA, France). The placebo was composed of a non-drug gel vehicle. In addition, the pimple pin was also used in the study.

### Treatment Procedures

In the first week of treatment, the inflammatory mediators of patients were partly cleared up with the pimple pin by an experienced paramedic after cleaning and disinfecting the face with bromogeramine every day, which can avoid the spread of bacterial infection during the treatment effectively. Then, AVG combined with US (20 W, 50 Hz, and 220 V) was applied to the patients' faces for 10–15 min, after which the soft mask (2 mm thickness) was applied to these patients for 20–30 min, and their faces were then cleaned. After the first week, the patients received the combination treatment of US and soft mask three times a week for 7 weeks. The duration of treatment cycle was 8 weeks. The controls only received placebo composed of the non-drug gel vehicle after clearing up with bromogeramine and the pimple pin, and then the faces were cleaned three times a week for 8 weeks.

### Efficacy Endpoints

The number of papules/pustules and the area of hyperpigmented lesions were counted as described previously (21), performed by the same investigator for all patients. Photographs were taken before and after treatment. Skin roughness and red area (an indicator reflecting facial blood circulation) were detected within a quantitative framework by a smart mirror intelligent facial detection system (Langdy, LD6021D, China).

The severity of acne was studied before and after the whole therapeutic session, following more comprehensive evaluation criteria (Table 1) that integrated two systems, including the Investigator's global assessment scale (IGA) for acne severity and the PIH Severity Scale (22). Of note, the number of facial lesions is also very important to assess acne severity quickly. Thus, it was added to the grading score to subdivide the acne severity based upon the modified Pillsbury grading set by the Chinese Medical Association. The acne severity was divided into 4 degrees and 10 grades, and n grades were awarded n scores. After treatment, the severity of acne in the same area was assessed by the same criteria. The score before treatment (sB) and the score after treatment (sA) were recorded and utilized to calculate the efficacy index (EI): EI = (sB-sA)/sB × 100%. The efficacy criteria included four grades: clinical cure (EI > 95%), markedly effective (EI: 60–95%), effective (EI: 20–59%), and invalid (EI < 20%).

**Table 1:** Investigator's assessment scale (IGA) for acne severity combined with post-inflammatory hyperpigmentation severity.

	**Definition**	**Grade**	**Number of papules and pustules (half of the face)**	**Pigmentary intensity of hyperpigmented lesions**	**Area of hyperpigmented lesions (% facial area)**
Clear or almost clear	Residual hyperpigmentation and erythema may be present. A few scattered comedones and a few small papules.	0	<10	None or trace	0–10%
Mild	Easily recognizable; less than half the face is involved. Some comedones and some papules/pustules.	1 2 3	10–25 26–50 51–75	Mild (localized)	11–25%
Moderate	More than half of the face is involved. Many comedones, papules/pustules. One nodule may be present.	4 5 6	76–100 >100 With 0 or 1 nodules or cysts	Moderate (diffused)	26–50%
Severe	Entire face is involved. Covered with comedones, papules/pustules. Presence of nodules/cysts.	7	With 2–4 nodules or cysts	Severe (prominent)	>50%

### Statistical Analysis

The results were analyzed on an intention-to-treat basis, and all statistical analyses were performed with SPSS 24.0 software (SPSS Inc., Chicago, IL, USA). Based on the normal distribution, the data are expressed as the mean ± SD and were analyzed with unpaired *t*-test between two groups. All statistical tests were two-sided, with a significance level of 0.05.

## Results

### Comparison of the Average Number of Papules and Hyperpigmented Area Before and After Treatment in the Two Groups

**Figure 1:**
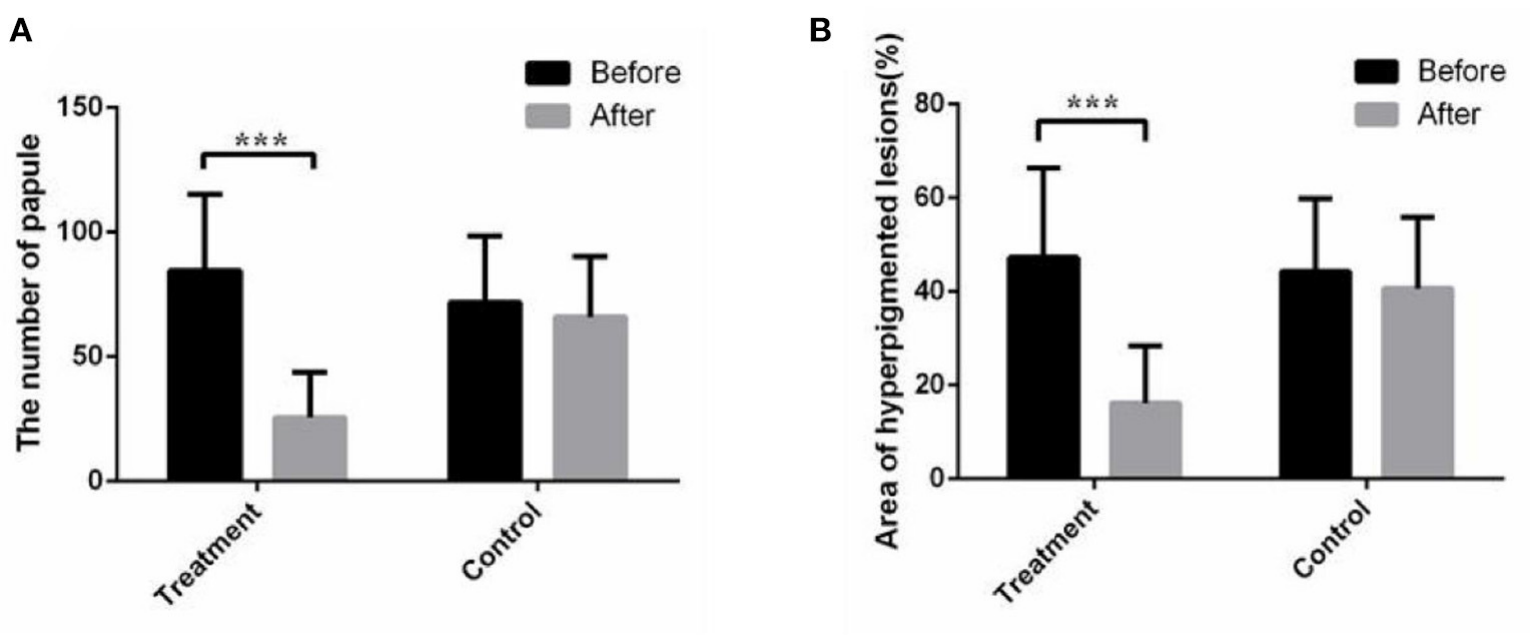
The combined therapy reduced the number of papules and facial hyperpigmented area in the treatment group. **(A)** Average total number of papules/pustules before and after treatment in the treatment group and control group. **(B)** Average facial hyperpigmented area (%) before and after treatment in the treatment group and control group. The results are expressed as the mean ± SD. ***p < 0.001.

**Figure 2:**
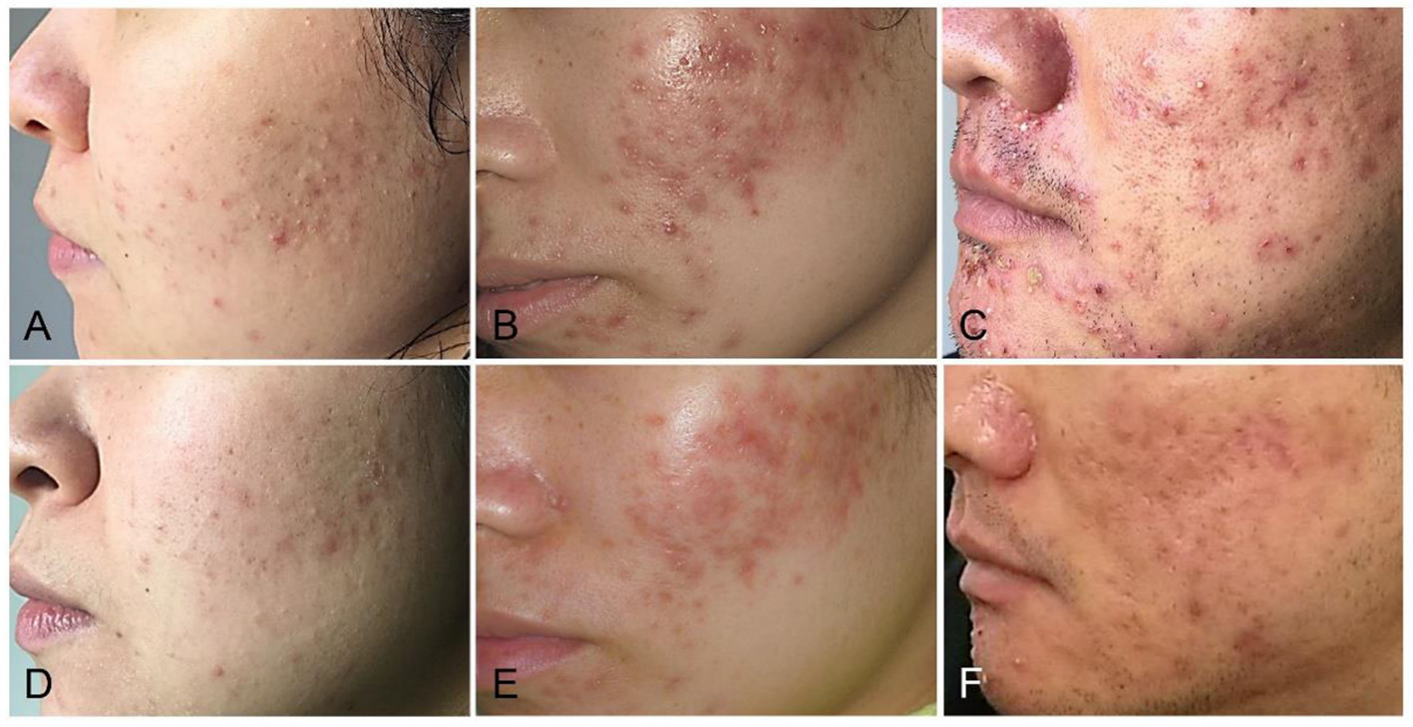
The combined therapy improved the acne condition in patients with mild to severe acne. **(A,D)** Representative photographs of mild acne patients before and after 2 months of the combined treatment. **(B,E)** Representative photographs of moderate acne patients before and after 2 months of the combined treatment. **(C,F)** Representative photographs of severe acne patients before and after 2 months of the combined treatment.

The papules/pustules and pigmentary intensity are the most common symptoms of acne. The results showed that the number of facial papules and hyperpigmented area (%) were 84.50 ± 30.78 and 47.28 ± 19.09 in the treatment group before the combined therapy, respectively. However, the number of facial papules were reduced to 25.53 ± 18.11 (*p* < 0.01) and the hyperpigmented area (%) was decreased to 16.15 ± 12.25 (*p* < 0.01) (Figure 1A), after the 2-month combined therapy. The skin conditions were significantly improved with treatment, as the number of facial papules/pustules were significantly decreased, and the area of hyperpigmented lesions was markedly reduced (Figure 2). However, no obvious improvement was observed in the control group after 2-month placebo treatment composed of the non-drug gel vehicle and face cleansing (*p* > 0.05) (Figure 1B).

### Comparison of Facial Skin Blood Circulation Before and After Treatment in the Two Groups

**Figure 3:**
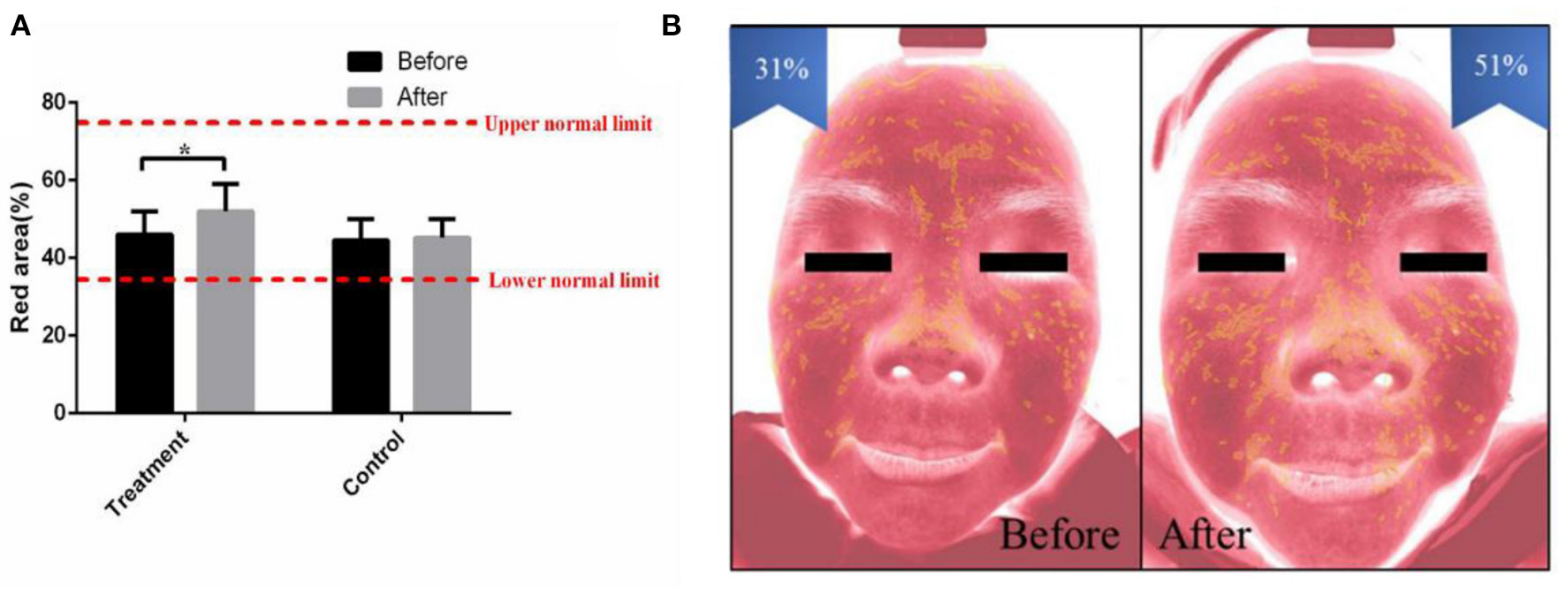
The combined therapy decreased the red area in the treatment group. **(A)** The proportion of red area before and after treatment in the treatment group and control group. **(B)** Representative pictures of a 27-year-old female taken by intelligent facial detection in the treatment group, which shows the comparison between the red area values before and after treatment. The results are expressed as the mean ± SD. *p < 0.05.

The distribution of capillaries and the capability of blood circulation are closely related to acne (23). In this study, the proportion of the red area was used to reflect the cutaneous blood circulation detected by intelligent facial detection. The increased red area indicated improved blood circulation. The results showed that the proportion of facial red area increased obviously after 2 months of combined therapy in the treatment group (*p* < 0.05), while there was no difference in the proportion of facial red area in the control group (Figure 3).

### Comparison of Facial Skin Roughness Before and After Treatment in the Two Groups

**Figure 4:**
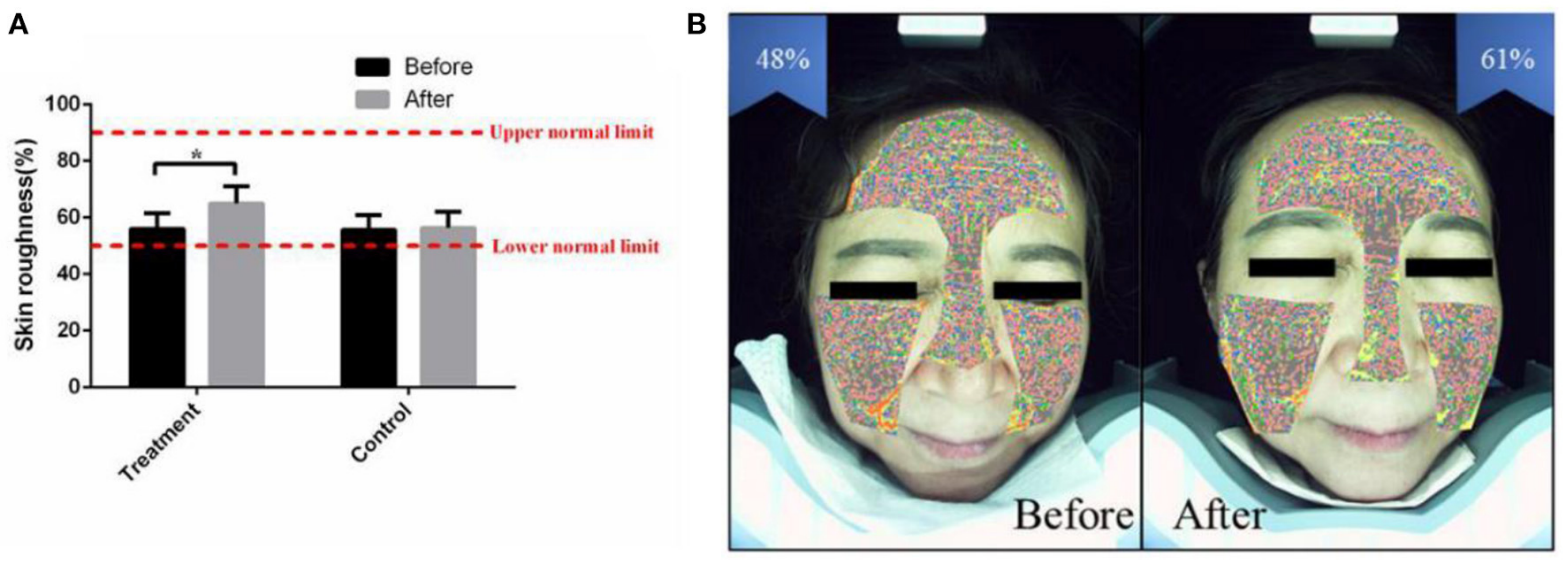
The combined therapy rescued the skin roughness in the treatment group. **(A)** The average value of facial skin roughness (%) before and after treatment in the treatment group and control group. **(B)** Representative pictures of a 32-year-old female taken by intelligent facial detection in the treatment group, which shows the comparison between the values of facial skin roughness before and after treatment. The results are expressed as the mean ± SD. *p < 0.05.

Facial skin roughness is based on the percentage of texture thickness, surface protruding area and pore distribution area, which indicates the current facial smoothness (24). Intelligent facial detection showed that the average facial skin roughness (%) of the treatment group was 56.20 ± 5.50 before receiving the combined therapy. After 2 months of combined therapy, the average facial skin roughness (%) was increased to 65.12 ± 6.20 (*p* < 0.05) (Figure 4A). After the combined treatment, the skin surface became soft and smooth, and the uncomfortableness induced by rough skin was markedly eased (Figure 4B). The facial skin roughness of the control group was similar to that 2 months prior (Figure 4A).

### Efficacy Evaluation After Treatment in Two Groups

In this study, the skin conditions of 24 patients in the control group and 40 patients in the treatment group before and after treatment were classified and evaluated after 2 months of treatment. The therapeutic efficacy data are shown in Table 2.

**Table 2:** The therapeutic efficacy of acne vulgaris in two groups.

**Group**	**Degree**	**Case number**	**Markedly effective case**	**Effective case**	**Invalid case**	**Average efficacy index**
Treatment group	Mild	6	0	6	0	40.00%
	Moderate	26	24	2	0	72.05%
	Severe	8	0	8	0	30.21%
	Total	40	24	16	0	-
Control group	Mild	5	0	1	4	6.67%
	Moderate	12	0	2	10	9.31%
	Severe	7	0	1	6	7.14%
	Total	24	0	4	20	-

**Figure 5:**
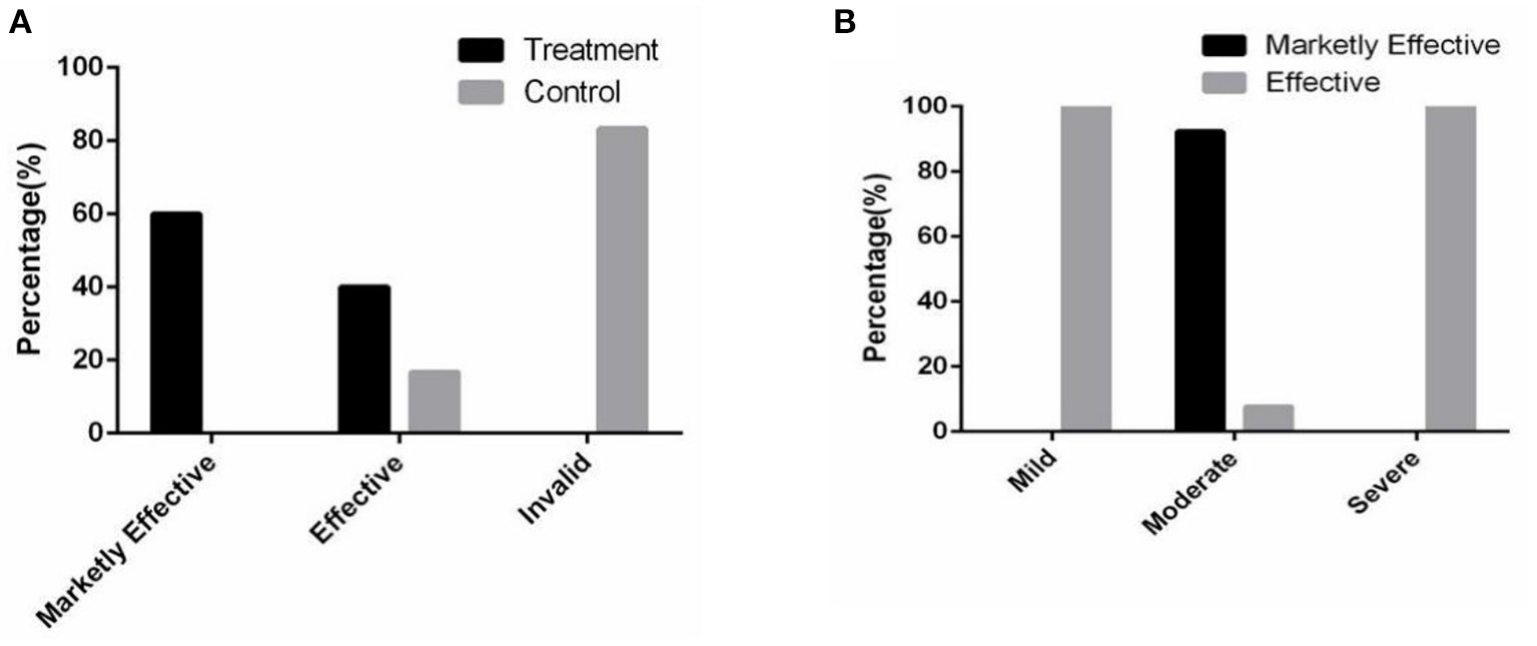
The combined therapy significantly promoted the skin condition in the treatment group. **(A)** The proportion distribution of the total efficacy in two groups. **(B)** The proportion distribution of the efficacy in the treatment group.

The results showed that in the treatment group, the effective rate was 40.00%, and the marked effective rate was 60.00% after 2 months of combined therapy. In the control group, the effective rate was only 16.67%, and the invalid rate was 83.33% (Figure 5A). Of note, the markedly effective rate of the patients with moderate acne in the treatment group was 92.31% (Figure 5B). In addition, the values of the average efficacy index of the patients with mild, moderate and severe acne were 40.00, 72.05, and 30.21%, respectively (Table 2). Of note, the patients with moderate facial acne had the highest average efficacy index, indicating that the combined therapy had its best effect on these patients.

### Safety Evaluation in the Course of Treatment in the Treatment Group-

**Figure 6:**
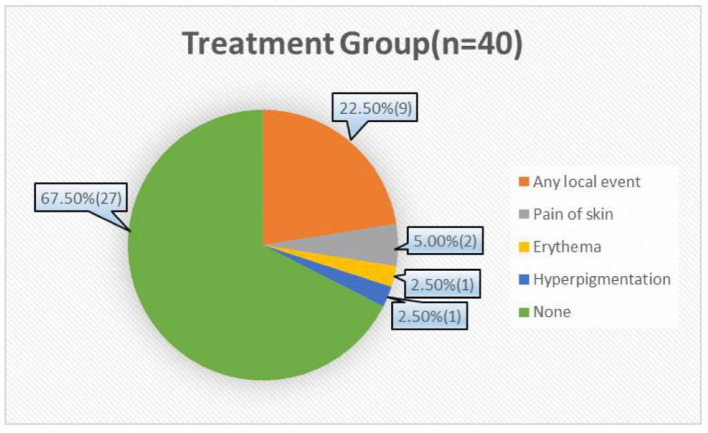
Side effects occurred during the course of treatment in the treatment group.

In this study, no serious side effects occurred during the whole treatment cycle in the treatment group and the control group. In the treatment group, nine patients had mild local reactions, such as itching, local inflammation, and scattered small abscesses. Two patients complained of mild pain at the treatment site. One patient had a small area of erythema in the treatment site, and one patient had a slight increase in facial pigmentation (Figure 6). However, these symptoms were tolerable and had been improved at the end of the treatment. There were no side effects, such as dry skin, chapped skin, gastrointestinal reaction, dizziness, or nausea, which were observed during the process of traditional acne treatment. Due to the properties of pure physical therapy, the possibility of drug resistance, and teratogenicity in the future may be very low.

## Discussion

To the best of our knowledge, the therapy that combines grease removal and AVG importing by ultrasonic and soft mask is the first non-drug treatment of acne. The current treatments of acne usually utilize oral and/or topical medications, which are mainly antibiotics and retinoids (8, 25). However, it was widely reported that retinoids treatment led to highly frequent adverse irritation, such as erythema, dryness, stinging, and burning (9, 26). Besides, although skin lesions subside after acne treatment, inflammatory pigmentation easily develops in the later stage of inflammation due to the photosensitivity of these drugs. It extends the time to repair skin significantly and affects the appearance of patients, leading to a great psychological burden to patients. Moreover, many other adverse reactions, including dizziness, nausea, and gastrointestinal reaction, excruciate acne patients in the course of treatment (27, 28). In terms of the application of antibiotics, its extensive use has inevitably resulted in an alarming rise in Propionibacterium acne resistance, thus obviously weakened effects (29). Our observations demonstrated that the new combined therapy had a very significant anti-inflammatory effect, pigmentation reduction and post-inflammatory acne recovery with fewer adverse reactions than previous treatments. In addition, it improved the skin conditions by replenishing cutaneous moisture and smoothing the skin.

At present, researchers have not yet reached a consensus on the severity scale and therapeutic efficacy of acne vulgaris globally. Because IGA for acne severity does not involve facial pigmentation or evaluate the acne condition quantitatively, the PIH severity scale and the number of facial lesions were incorporated into IGA (22). The new evaluation system we proposed is more comprehensive and practical.

Acne is caused by chronic inflammatory lesions of pilosebaceous units. Hyperkeratosis and sebum blocking pores are the earliest changes (30). If irritable free fatty acids are not cleared, they will continue to stimulate hair follicles, leading to facial hyperpigmentation in post-inflammatory acne. Thus, the inflammatory papules and pustules were treated with the pimple pin first to reduce the sustained skin damage caused by the accumulation of sebum. In addition, the repatency of pilosebaceous ducts will benefit AVG permeation. Although the pimple pin may damage part of the skin tissue, slight injury to human skin has no significant effect. Of note, before ultilizing the pimple pin, bromogeramine was used to disinfect the skin without irritation, avoiding the possible infection during the treatment.

As a traditional natural medicine, AVG possesses a variety of biological effects, including anti-inflammatory, antioxidative, and antipigmentation effects, which are beneficial for acne treatment and repair of skin lesions (31, 32). The neutral polysaccharides, aloe mannan and acemannan separated from the gel part of aloe leaves have been identified to have anti-inflammatory and immunosuppressive effects by reducing the adhesion of leukocytes and the level of TNF-α and ameliorating the capability of self-repair by inhibiting thromboxane (an inhibitor of lesion repair) (33–35). The oxidation reaction of skin lesions is also one of the main reasons that prolongs the process of lesion repair. Glutathione peroxide activity, superoxide dismutase enzymes, and phenolic antioxidants were found to be present in AVG, which may be responsible for the antioxidant effects (36). AVG can inhibit tyrosinase and dihydroxyphenylalanine (DOPA), a priming agent in the enzymatic conversion of tyrosine into melanin that reduces pigmentation (37). The muco-polysaccharides along with amino acids and zinc found in aloe vera are capable of maintaining the integrity and moisture of skin, reducing erythema and helping prevent skin ulcers (38, 39). In addition, studies have also shown that AVG is capable of increasing the flexibility of skin and reducing its fragility (40). Furthermore, as a natural substance, AVG is easier to obtain and has fewer side effects than chemical medicines.

To enhance the absorption effect, AVG was imported into the epidermis by ultrasound. The physical and chemical properties of ultrasound were identified as useful for drug depolymerization and lowering viscosity (41). In addition, it was also found that ultrasound could stimulate the decomposition of the stratum corneum and promote the convective motion of intraepidermal molecules (42). Thus, ultrasound can enhance the absorption of drugs, nutrients, and cream. Moreover, the mechanical action and thermal effect of ultrasound are useful to promote cutaneous blood circulation and increase the permeability of the cell membrane, thereby enhancing tissue metabolism and regeneration capability (43). Meanwhile, the microcirculation and lymphatic circulation of skin will be accelerated by ultrasonic irradiation, which improves the absorption and removal of necrotic tissue. Because of the skin's barrier function, the absorption rate of topical medicine is not sufficient. Thus, based on the features of ultrasound, AVG penetrates percutaneously into the lesions more easily to enhance the skin's self-repair capabilities. In addition, there are several effective mechanisms in the treatment of topical lesions, including inhibition of bacterial colonization, subsequent tissue formation, neovascularization, and macrophage stimulation for fibroblast proliferation (44). According to previous reports, various ultrasound frequencies, intensities and treatment durations will result in various effects. The most common ultrasound treatment of inflammatory conditions is low-intensity, high-frequency and pulsed mode for minimizing thermal effects, whereas, the optimal protocol of ultrasound-assisted repair of skin lesions will be further explored in future studies (45).

A moist membrane can be formed on the skin surface by using a soft mask, thereby protecting skin from the external environment and achieving transient softening and fat gettering. In addition, the soft mask has a role in pore cleansing, anti-inflammation and moisture-oil balance. Therefore, using soft mask is not only for a more effective therapeutic effect but also for the prevention of acne regeneration.

It is not the first time to apply AVG or US in the researches related with acne treatment. However, their therapeutic effects were not satisfactory. A study has confirmed that the cream containing propolis, tea tree oil and AVG was effective in reducing acne, however, the role of which was restricted to the decreased number of papules/pustules and erythema without the evaluation of the pigmentation improvement and the overall acne condition (46). In addition, the use of US contributed to the antibacterial effects of lysozyme-shelled microbubbles on inhibiting the inflammation of acne, but the results of which were limited and lacked the comparisons of other important clinical features associated with acne treatment (47). Of note, it has been widely recognized that personal lifestyle and diet affect the development of acne. For example, sleep duration and quality have a reciprocal relationship with acne occurrence and severity (6). A lot of evidence indicated that high glycemic load diet could exacerbate acne (48). Thus, during the treatment, our patients were encouraged to avoid greasy and high glucose diet, wash their faces regularly, maintain a regular schedule, etc., In the absence of these possible influence factors, it is more conducive to assess the curative effect of the combine therapy.

In our study, the combined therapy lasted 2 months followed by a 1-month follow-up period, during which it was observed that the hyperpigmentation condition was further improved in the treatment group, indicating that the 2-month combined therapy conduced to post-inflammatory recovery. However, a limitation of the study is the short-term follow-up period, which should be extended from 1 to 3 months even longer to collect more long-term results and assess the possible recurrence rate.

In summary, the non-drug acne therapy we proposed, which combined grease removal and AVG importation by ultrasonication and soft mask, can accelerate the recovery of post-inflammatory acne. The therapeutic effect is the most prominent for patients with moderate acne. As a non-drug therapy, it has more advantages than traditional therapies, such as more obvious effects, easier operation, and safer experience. Despite the relatively long treatment cycle of the combined therapy, fewer side effects are assured due to its properties of pure physiotherapy. Most people may be more willing to accept safer treatment without sequelae than to replace the current damage with other losses. Therefore, this new treatment is worthy of further promotion and application.

## Data Availability Statement

The raw data supporting the conclusions of this article will be made available by the authors, without undue reservation.

## Ethics Statement

The studies involving human participants were reviewed and approved by the Ethics Committee of Army Medical University. The patients/participants provided their written informed consent to participate in this study. Written informed consent was obtained from the individual(s) for the publication of any potentially identifiable images or data included in this article.

## Author Contributions

HZ carried out the experiments, collected and analyzed the data, and drafted the manuscript. WZ, XS, and XL contributed to acquisition and data analysis. HL and YL designed the experiments, supervised the project, and revised the manuscript. All authors contributed to the article and approved the submitted version.

## Conflict of Interest

The authors declare that the research was conducted in the absence of any commercial or financial relationships that could be construed as a potential conflict of interest.
